# Diagnostic Performance of Digital Tomosynthesis for Detecting Osteophytes and Subchondral Bone Changes in the Equine Distal Interphalangeal and Metacarpophalangeal Joints

**DOI:** 10.3390/ani16182971

**Published:** 2026-09-21

**Authors:** Alisa Hahn, Miguel Valdés Vázquez, Gabriel Manso-Díaz, Dagmar Berner, Joana Maia Ramos, Marcus G. Doherr, Christoph J. Lischer

**Affiliations:** 1 Equine Clinic, Veterinary Hospital, School of Veterinary Medicine, Freie Universität Berlin, Oertzenweg 19b, 14163 Berlin, Germany; christoph.lischer@fu-berlin.de; 2 Hospital de Referencia La Equina, Apdo. 110, Camino de Martagina Km 1, 29692 Manilva, Málaga, Spain; miguel@laequina.com (M.V.V.); joana.126@hotmail.com (J.M.R.); 3 Department of Animal Medicine and Surgery, Faculty of Veterinary Medicine, Complutense University of Madrid, Avenida Puerta de Hierro, s/n, 28040 Madrid, Spain; gmanso@ucm.es; 4 Equine Referral Hospital, Department of Clinical Science and Services, Royal Veterinary College, Hawkshead Lane, North Mymms, Hatfield, Hertfordshire AL9 7TA, UK; dberner@rvc.ac.uk; 5 Institute of Veterinary Epidemiology and Biostatistics, School of Veterinary Medicine, Freie Universität Berlin, Königsweg 67, 14163 Berlin, Germany; marcus.doherr@fu-berlin.de

**Keywords:** digital tomosynthesis, computed tomography, osteoarthritis, osteophytes, subchondral bone, musculoskeletal imaging, sensitivity and specificity, ex vivo

## Abstract

Osteoarthritis is a common cause of equine lameness and can involve periarticular osteophytes and subchondral bone (SB) changes. This exploratory ex vivo study evaluated the diagnostic performance of digital tomosynthesis (DT) for detecting these findings in the distal interphalangeal and metacarpophalangeal joints, using computed tomography (CT) classifications as the reference. Sixteen distal forelimbs from nine adult horses underwent imaging with both modalities under simulated weight-bearing conditions. Three independent observers graded osteophytes and SB changes, and the scores were dichotomised as absent or present for analysis. The primary analysis used a majority-based CT reference, defined by agreement of at least two observers. Observer-specific comparisons and within-observer DT–CT agreement were evaluated in a secondary analysis. In the primary analysis, sensitivity ranged from 23.1% (95% CI 9.5–39.3%) to 34.6% (95% CI 13.8–56.0%), specificity from 94.1% to 96.1%, Youden’s index from 19.2% to 30.7%, and Cohen’s κ from 0.249 to 0.377. Sensitivity was numerically higher for osteophytes than for SB changes for all three observers, without formal comparison. DT missed many CT-positive assessments but produced few false-positive classifications. Studies in live horses comparing DT with conventional radiography are needed to determine its additional diagnostic value.

## Simple Summary

Osteoarthritis frequently affects joints in horses. Small bony growths around joints and changes in the bone beneath the joint cartilage can occur with osteoarthritis. This study investigated how well digital tomosynthesis, a technique that produces a series of sectional X-ray images, could identify abnormalities in the coffin and fetlock joints using computed tomography as the reference imaging method. Sixteen lower forelimbs collected after death from nine horses were examined under simulated weight-bearing conditions using both imaging methods, and three veterinarians evaluated the images independently. An abnormality was considered present on computed tomography when at least two of the three observers identified it. Depending on the observer, digital tomosynthesis detected 23.1–34.6% of abnormalities identified using this reference and therefore missed many of them. It rarely indicated an abnormality when the computed tomography reference indicated none. All three observers detected a greater proportion of bony growths around joint margins than changes beneath the cartilage, although these proportions were not formally compared. Studies in live horses and direct comparisons with conventional radiography are needed to determine whether digital tomosynthesis provides additional diagnostic information in clinical practice.

## 1. Introduction

Osteoarthritis (OA) is one of the leading causes of lameness in horses [1,2], particularly in athletic animals [3,4,5]. Conventional radiographic assessment of OA is based primarily on the identification of osseous abnormalities, including osteophyte formation and subchondral bone (SB) remodelling [2,6], whereas early cartilage degeneration remains largely invisible [7]. The distal interphalangeal (DIP) [8,9,10,11,12] and metacarpophalangeal (MCP) [2,13,14] joints are commonly affected by OA.

Conventional radiography remains the primary imaging modality for evaluating osseous changes associated with OA in equine joints because it is widely available, relatively inexpensive, and can be performed in standing horses under weight-bearing conditions [15,16,17]. However, its diagnostic performance is limited by the two-dimensional nature of the technique and the resulting superimposition of complex anatomical structures [10,18,19].

Cross-sectional imaging largely overcomes these inherent limitations [19,20]. Computed tomography (CT) provides high spatial resolution for osseous structures and has become an established imaging modality for the assessment of osteophytes, subchondral bone sclerosis, cyst-like lesions, and other osseous abnormalities associated with OA [19,20,21]. Although standing CT systems now permit distal limb imaging without general anaesthesia, high equipment costs and restricted availability continue to limit routine clinical use [22,23,24]. Consequently, there remains a clinical need for an imaging technique that provides tomographic information beyond conventional radiography while retaining its practical advantages in the standing horse.

Digital tomosynthesis (DT) is an X-ray-based imaging modality that reconstructs multiple sectional images from a series of low-dose radiographic projections acquired over a limited angular range [25,26,27]. By reducing the superimposition of anatomical structures, DT can improve the visualisation of osseous abnormalities compared with conventional radiography [28], while generally involving lower radiation doses and costs than CT [29].

In horses, portable DT equipment is commercially available for imaging standing, minimally sedated animals, thereby allowing image acquisition under physiological weight-bearing conditions [28]. Because DT is an X-ray-based technique, it is particularly suited to the assessment of osseous abnormalities, including osteophytes and SB changes associated with OA [28,30]. Although DT has been evaluated in human musculoskeletal imaging and has demonstrated improved detection of osseous abnormalities compared with conventional radiography across several anatomical regions [29,30,31,32,33,34,35], evidence regarding its use in equine orthopaedics remains limited. To date, only one equine study has investigated DT for evaluating osteochondral lesions in cadaveric MCP joints and reported that DT may provide additional diagnostic information compared with conventional radiography [28]. However, the diagnostic performance of DT for identifying osteophytes and subchondral bone changes associated with equine OA has not yet been established.

Therefore, the aim of this study was to evaluate the diagnostic performance of DT for the detection of osteophytes and SB changes in the equine DIP and MCP joints, using multidetector CT as the reference imaging modality. We hypothesised that DT would show high specificity but limited sensitivity for detecting these abnormalities. A secondary objective was to estimate DT sensitivity separately for osteophytes and SB changes.

## 2. Materials and Methods

### 2.1. Specimens

The present analysis was considered exploratory. No separate sample size calculation was performed for the assessment of naturally occurring osteophytes and subchondral bone changes. Sixteen distal forelimbs (eight right and eight left) were included from nine adult horses: bilaterally from seven horses and unilaterally from two. Paired forelimbs from the same horse were evaluated separately. The horses had a mean age of 13 years (range 3–20 years) and a mean body weight of 528 kg (range 480–650 kg). The nine horses comprised four mares, four geldings, and one stallion. Breeds included Hanoverian, German Sport Horse, and Thoroughbred (*n* = 2 each), and Holsteiner, Arabian, and Pura Raza Española (*n* = 1 each). All limbs were obtained within 12 h of euthanasia at the Equine Hospital, Freie Universität Berlin. The horses were euthanised for reasons unrelated to the present study: seven because of severe gastrointestinal disease and two because of other non-orthopaedic conditions. Individual horse and limb characteristics are provided in Supplementary Table S1. The study protocol was approved by the local research ethics committee (StN 046/23, 16 January 2024).

Following collection, each limb was disarticulated at the middle carpal joint, with all surrounding soft tissues left intact. The limbs were individually labelled, wrapped in plastic film, and stored at temperatures below −20 °C. Before further processing, each limb was thawed separately at room temperature for approximately 12 h under the same conditions and was then cleaned and clipped. Horseshoes were removed where present.

### 2.2. Imaging Protocol

**Figure 1:**
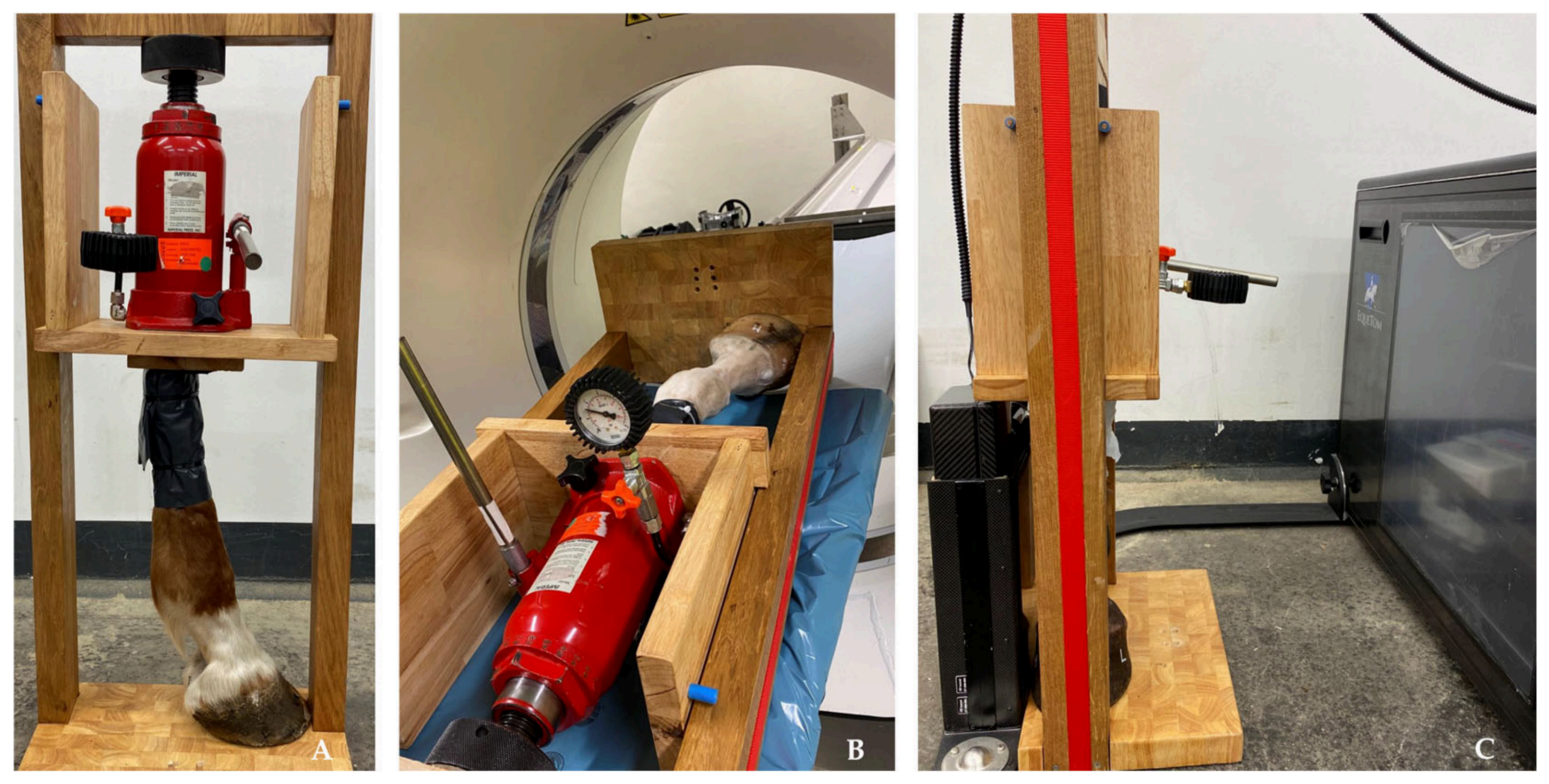
Experimental setup for simulated weight-bearing (SWB) during imaging. Axial load was applied to the proximal end of the limb using the hydraulic jack. Hydraulic pressure was monitored using the integrated pressure gauge. (**A**) Distal equine forelimb mounted in a wooden loading frame with an integrated hydraulic jack. (**B**) Loaded limb positioned within the computed tomography (CT) gantry. (**C**) Loaded limb positioned between the digital tomosynthesis (DT) detector and the X-ray tube housing for acquisition of a dorsopalmar projection.

The limbs were maintained at room temperature throughout the imaging procedures. Imaging under simulated weight-bearing (SWB) conditions was performed using a custom-built wooden loading frame measuring 115 cm × 40 cm × 26 cm (height × width × depth) and incorporating a hydraulic jack (Figure 1). The reference load of 1000 N per limb for a 450-kg horse reported in a previous ex vivo study [36] was scaled to the mean body weight of 528 kg, resulting in a calculated load of 1173 N. The hydraulic pressure measured by the gauge integrated into the jack was converted to axial force using F = pA and a piston cross-sectional area of 91.6 cm^2^, calculated from a piston diameter of 10.8 cm. The calculated load of 1173 N corresponded to a hydraulic pressure of 128 kPa (1.28 bar). For consistency, the same pressure-gauge reading was used for all limbs. The axial force was calculated from the measured hydraulic pressure and piston area but was not independently verified by direct measurement. For CT, the frame and limb were positioned horizontally, with the limb in lateral recumbency. Axial compression held the limb securely within the frame, and the pressure-gauge reading remained constant throughout image acquisition. The load was released during transfer between CT and DT and reapplied to the same pressure-gauge reading. For acquisition of the different DT projections, the load was released and the limb was rotated within the frame to prevent parts of the wooden frame from being superimposed on the anatomical region of interest. After each repositioning, the load was reapplied to the same pressure-gauge reading.

DT was performed using the EqueTom™ digital tomosynthesis system (Orimtech Ltd., Buffalo Grove, IL, USA), which incorporated an Iona2-RF-2430 dynamic digital detector manufactured by Teleoptic PRC Ltd. (Kyiv, Ukraine) and mechanical components manufactured by SPA Teleoptika Ltd. (Kyiv, Ukraine). The Poskom PXM-20BT X-ray unit moved within the gantry, while the detector, with a field of view of 244 mm × 304 mm and 16-bit digitisation, was positioned on the opposite side of the limb. During image acquisition, the X-ray unit moved through a limited angular range while the detector acquired 120 radiographic projections over 3.2 s. The angular sweep used for the study acquisitions was not available from the acquisition records. These projections were subsequently reconstructed using Orimtech’s proprietary Ace-Clubs software. The DT acquisitions were initially reconstructed into 340 slices. All DT stacks were prepared by the same investigator (A.H.) immediately after image reconstruction and before dataset randomisation and blinding. Only peripheral slices at the beginning and end of each stack that were visually judged to be too blurred for assessment were removed. The number of removed slices varied among datasets according to the extent of peripheral blurring and was not recorded. All three observers reviewed the same prepared stack for each acquisition and could scroll freely through all retained slices. Tomosynthesis acquisitions (81 kVp, 50 mAs) included lateromedial and dorsopalmar views of both the MCP and DIP joints. In total, four DT acquisitions were obtained per limb. The datasets were reconstructed with a slice thickness of 0.6 mm, an image matrix of 1520 × 1280 pixels, and an in-plane pixel spacing of 0.18 mm × 0.18 mm. An X-ray marker was placed on the lateral aspect of the limb to facilitate image orientation. 

CT of the distal limb was subsequently performed from the mid-metacarpal region to the hoof using an Aquilion/LB multidetector CT system (Toshiba Medical Systems Corporation, Otawara, Japan). The limbs were positioned in lateral recumbency on the scanner table and a cannula sheath was placed on the lateral aspect of each limb to facilitate image orientation. Helical scans were acquired at 120 kVp and 250 mA, with a rotation time of 0.75 s, a pitch of 0.688, and a collimation of 16 mm × 1.0 mm. Images were reconstructed using AIDR 3D Mild with bone and soft-tissue reconstruction kernels, a slice thickness of 1.0 mm, a reconstruction interval of 0.8 mm, a 240 mm × 240 mm field of view, a 512 × 512 matrix, and an in-plane pixel spacing of 0.468 mm × 0.468 mm.

### 2.3. Image Analysis

Image assessments were performed by two board-certified veterinary radiologists (GM-D and DB, both DipECVDI) and one equine clinician with 10 years of clinical experience and a focus on sports medicine and diagnostic imaging (JMR, DVM), who had previously used DT in clinical practice. To provide a common basis for DT image assessment, all observers completed a standardised familiarisation session using seven DT images not included in the study. The observers independently reviewed all CT and DT imaging datasets using the Horos DICOM viewer for the presence and severity of osteophytes and SB changes in the MCP and DIP joints. No restrictions were imposed on the use of standard image display functions for either modality, including zooming, scrolling, and adjustment of window width and level. Multiplanar reconstructions were also permitted for the CT datasets. Before image assessment, each CT and DT dataset was assigned a sequence number using a web-based random-number generator, resulting in a mixed sequence of datasets from both modalities. The datasets were reviewed according to this sequence. Observers could complete the image assessments in one or more reading sessions. No predefined washout period was used. The observers were blinded to the number, location, and severity of osseous changes before image evaluation.

The anatomical sites included in the assessment were predefined before image evaluation. An osteophyte was defined as a bony outgrowth arising at the joint margin and extending beyond the normal bone contour. SB changes were defined as focal or diffuse abnormalities of the subchondral bone plate or immediately adjacent trabecular bone. These included sclerosis, lysis and focal defects or irregularity of the SB plate. Isolated irregularity of the SB plate was sufficient for classification as an SB change. Osteophytes were assessed at the joint level. Each DIP joint and each MCP joint was treated as one assessment target, and multiple osteophytes within the same joint did not generate additional observations. Similarly, each predefined anatomical site assessed for SB changes was treated as one assessment target, irrespective of the number of abnormalities present at that site.

For the DIP region, the following anatomical sites were evaluated: osteophytes of the DIP joint; SB changes in the middle phalanx (P2); SB changes in the distal phalanx (P3); and SB changes at the dorsal border of the navicular bone. For the MCP joint, the following anatomical sites were evaluated: osteophytes of the MCP joint; SB changes in the proximal phalanx (P1); SB changes in the third metacarpal bone (MC3); and SB changes in the proximal sesamoid bones.

**Figure 2:**
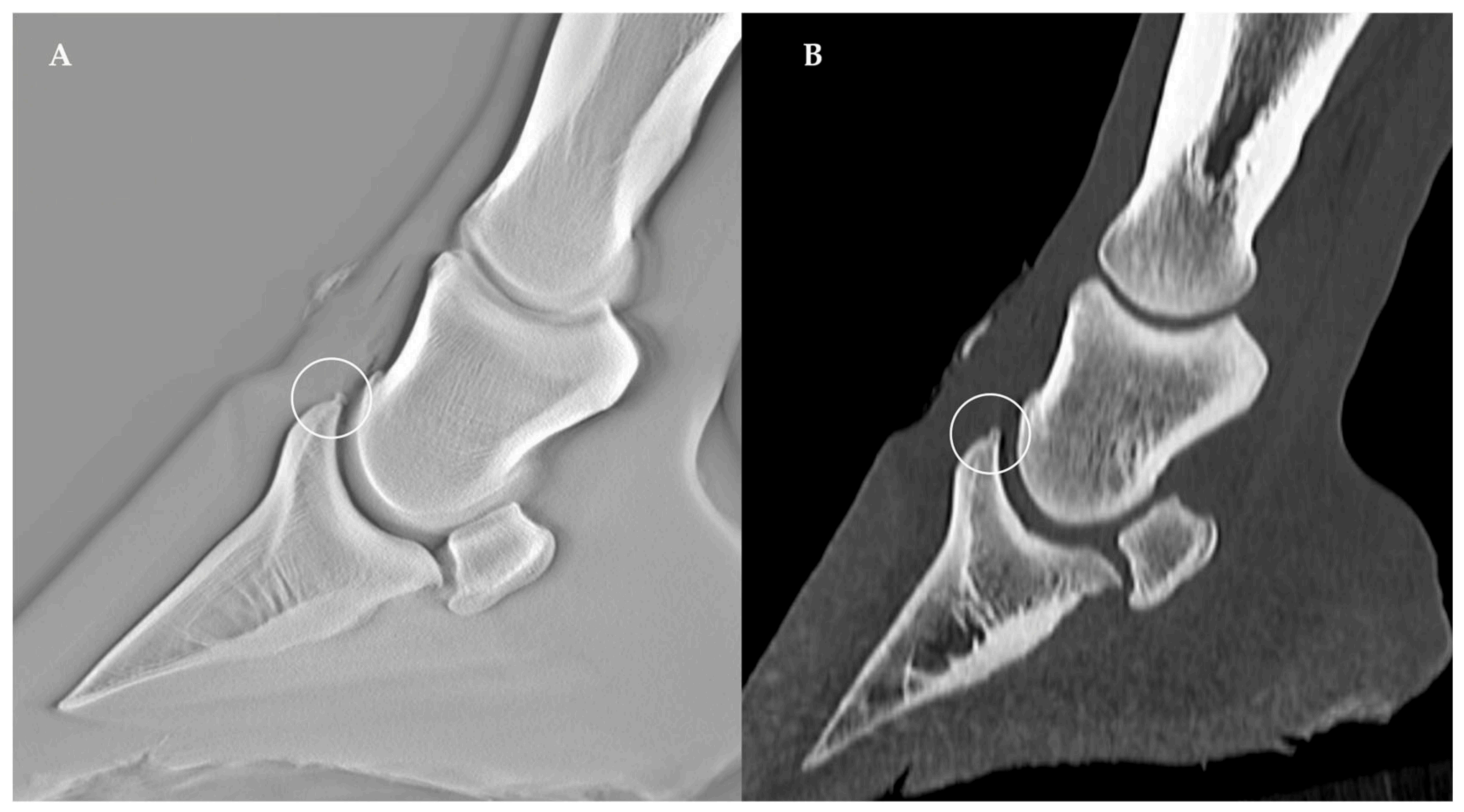
Comparison of digital tomosynthesis (DT; (**A**)) and computed tomography (CT; (**B**)) images of the distal interphalangeal (DIP) joint obtained under SWB conditions. Both modalities demonstrate an osteophyte at the proximal aspect of the extensor process of the distal phalanx (P3), highlighted by the white circles. All three observers detected the osteophyte on both modalities, assigning severity scores of 1–2 on DT and 2–3 on CT. The dorsal aspect is to the left.

**Figure 3:**
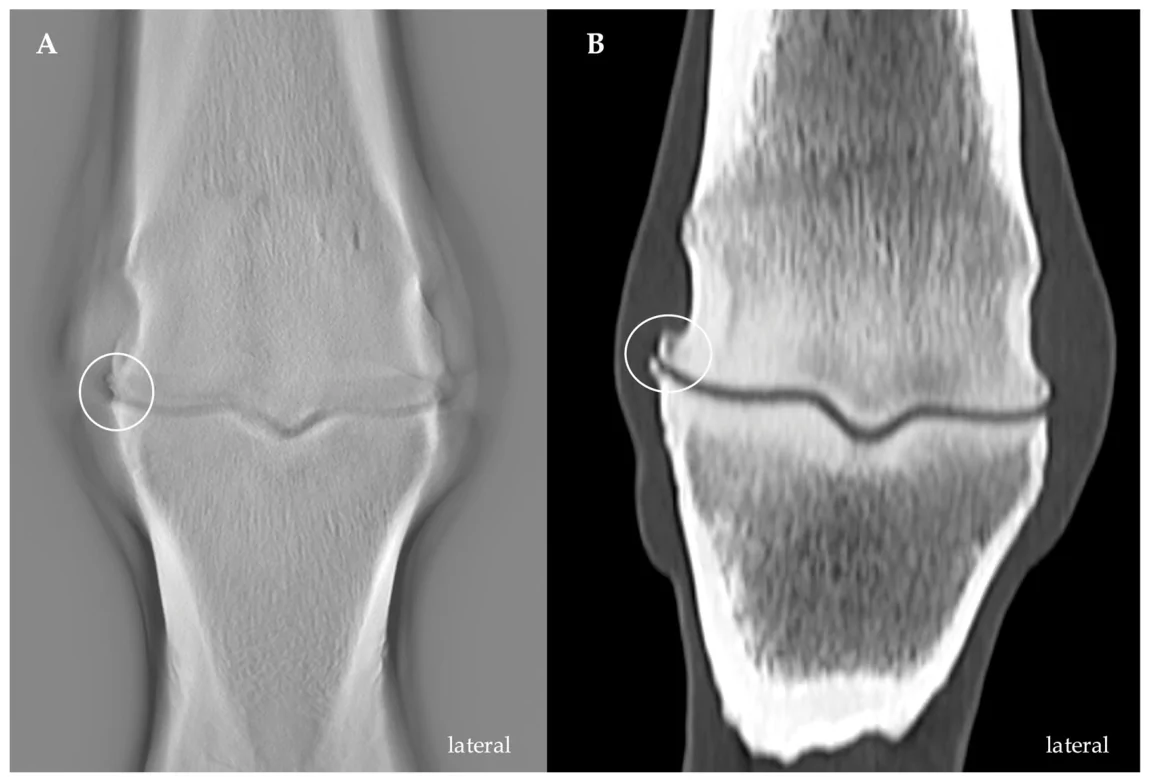
Comparison of DT (**A**) and CT (**B**) images of the metacarpophalangeal (MCP) joint obtained under SWB conditions. Both modalities demonstrate osteophyte formation along the medial articular margins of the proximal phalanx (P1) and the third metacarpal bone (MC3), highlighted by the white circles. All three observers detected the osteophyte formation on both modalities, assigning a severity score of 2 on DT and scores of 2–3 on CT. The lateral aspect is marked in both images.

**Figure 4:**
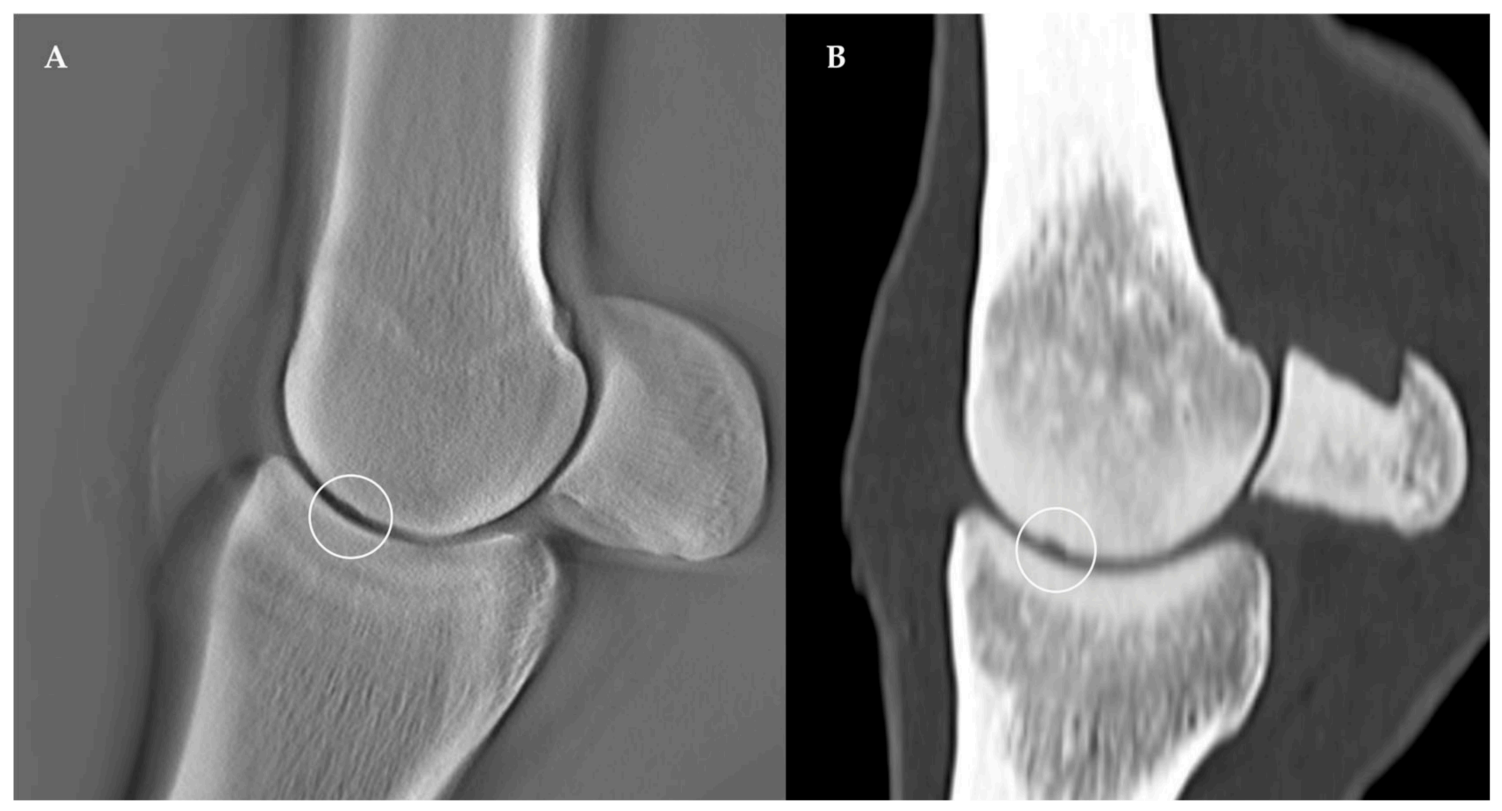
Comparison of DT (**A**) and CT (**B**) images of the MCP joint obtained under SWB conditions. Both modalities demonstrate a subchondral bone (SB) lesion at the distal aspect of MC3, highlighted by the white circles. The lesion is characterised by focal irregularity of the articular surface with an underlying focal radiolucency on DT and corresponding hypoattenuation on CT. Observer 1 recorded no change on either modality, Observer 2 recorded SB changes on both modalities, and Observer 3 recorded it only on CT. Across observers, severity scores ranged from 0 to 1 on DT and from 0 to 2 on CT. The dorsal aspect is to the left.

**Figure 5:**
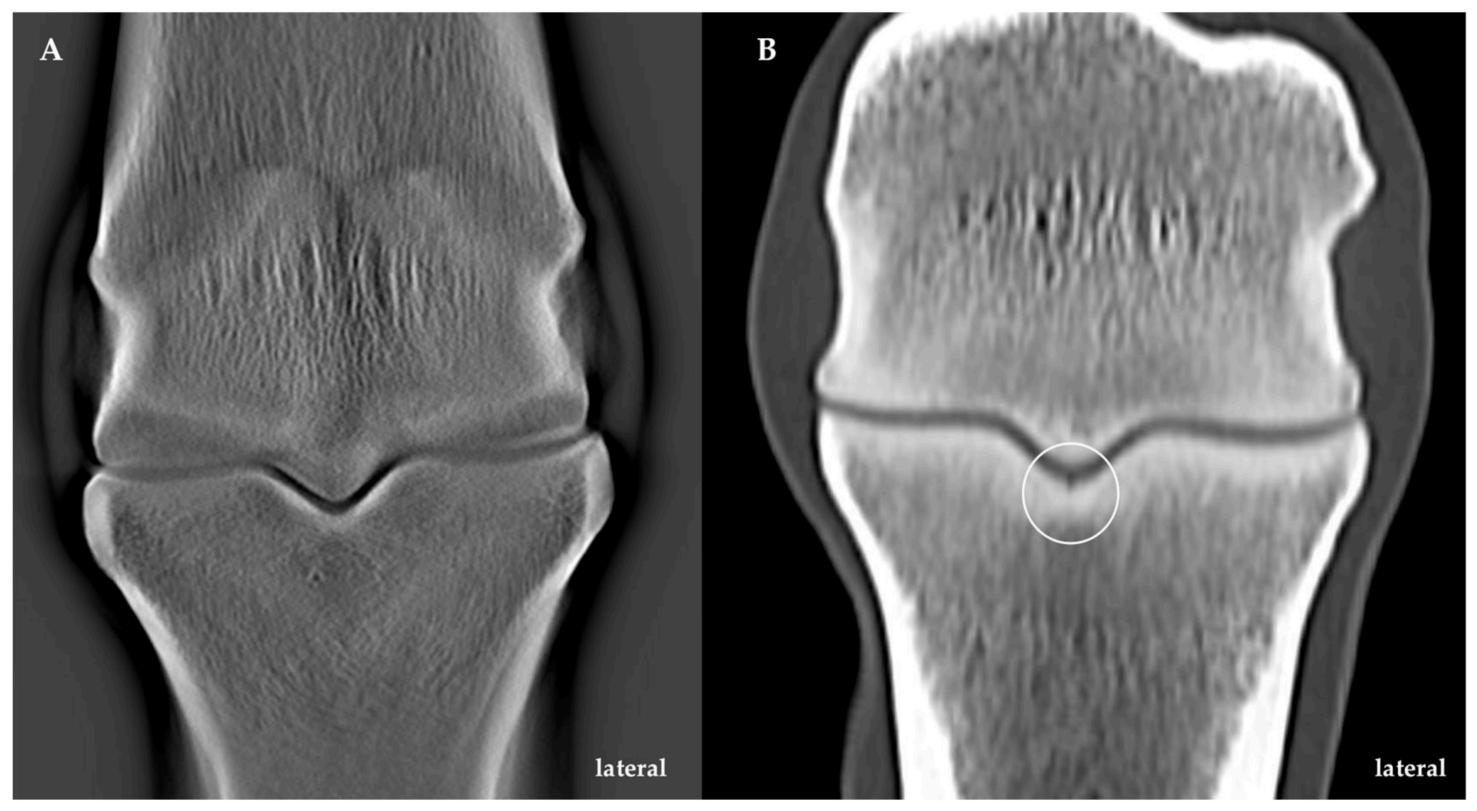
Comparison of DT (**A**) and CT (**B**) images of the MCP joint obtained under SWB conditions. CT demonstrates a fine fissure line extending distally from the sagittal groove of P1 with mild adjacent subchondral sclerosis, highlighted by the white circle. All three observers assigned a score of 0 on DT and severity scores of 1–2 on CT. The lateral aspect is indicated in both images.

**Figure 6:**
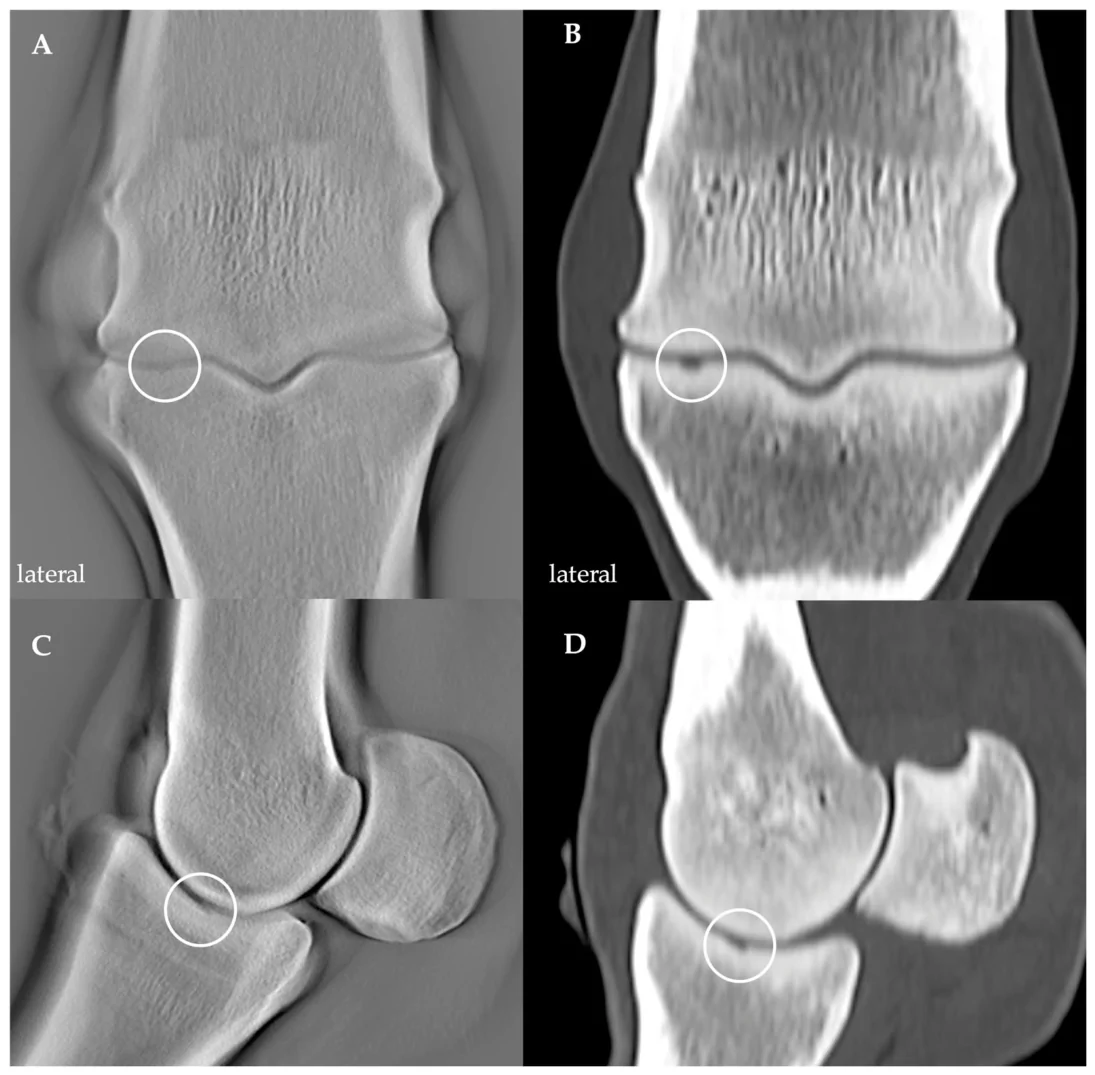
Comparison of DT (**A**,**C**) and CT (**B**,**D**) images of the MCP joint obtained under SWB conditions. Both modalities demonstrate an SB lesion at the proximolateral aspect of P1, highlighted by the white circles. The lesion is characterised by focal irregularity of the articular surface and an associated subchondral defect, which is radiolucent on DT and hypoattenuating on CT. Observer 1 did not detect the lesion on either modality, Observer 2 detected it only on CT, and Observer 3 detected it on both modalities. Across observers, severity scores ranged from 0 to 1 on DT and from 0 to 2 on CT. The lateral aspect is indicated in images (**A**,**B**). The dorsal aspect is to the left in images (**C**,**D**).

For each assessment target, each observer graded osseous changes using a four-point scale: 0 = no change, 1 = mild, 2 = moderate, and 3 = severe. No quantitative thresholds were predefined for differentiating between the severity grades. Representative examples of osteophytes and SB changes identified on DT and CT are shown in Figure 2, Figure 3, Figure 4, Figure 5 and Figure 6.

### 2.4. Statistical Analysis

The results of the blinded image assessments were entered into a decoded master spreadsheet in Microsoft Excel (Microsoft Corporation, Redmond, WA, USA). Statistical analyses were performed using IBM SPSS Statistics version 29.0 (IBM Corp., Armonk, NY, USA) and StataNow/SE 19.5 (StataCorp LLC, College Station, TX, USA).

For each observer and imaging modality, each limb contributed eight predefined anatomical assessments, yielding 128 assessments per observer and modality (16 limbs × 8 anatomical sites). These comprised 32 osteophyte assessments (16 limbs × 2 joints) and 96 assessments of SB changes (16 limbs × 6 anatomical sites). Across the observers, this yielded 384 individual ratings per imaging modality. For the diagnostic performance analyses, the scores were dichotomised to indicate the absence or presence of the respective finding. A score of 0 (‘no change’) was classified as absent, whereas scores of 1 (‘mild’), 2 (‘moderate’), and 3 (‘severe’) were classified as present. Diagnostic performance was not evaluated separately by lesion severity because of the small numbers of ratings in some severity categories. For the overall diagnostic performance analysis, data from all 128 anatomical assessments were combined for each observer to provide a descriptive summary across both joints.

In the primary analysis, a majority-based CT reference was derived for each anatomical assessment. An assessment was classified as positive when at least two of the three observers recorded the finding as present on CT and as negative when at least two recorded it as absent. Each observer’s dichotomised DT classification was compared with this majority-based CT reference. Sensitivity, specificity, accuracy, Youden’s index, and Cohen’s kappa (κ) were calculated separately for each observer across all 128 assessments. Youden’s index was calculated as sensitivity + specificity − 1, with sensitivity and specificity expressed as proportions, and reported as a percentage.

The secondary analysis was observer-specific. For each predefined assessment target, the dichotomised DT classification was compared with the dichotomised CT classification assigned by the same observer. Again, sensitivity, specificity, accuracy and Youden’s index of DT relative to CT were calculated separately for Observers 1, 2 and 3. Within-observer agreement between the dichotomised DT and CT classifications was assessed using Cohen’s kappa (κ).

Under the majority-based reference, sensitivity was calculated separately for osteophytes and SB changes. All predefined anatomical assessment sites were included. Sensitivity estimates were reported with the corresponding numbers of true-positive and CT-positive assessments (n/N) and 95% confidence intervals.

Interobserver agreement among the three observers was assessed separately for CT and DT. Fleiss’ κ and Gwet’s AC1 were calculated separately for the binary classifications of osteophytes (32 assessments) and SB changes (96 assessments), with score 0 classified as lesion absent and scores 1–3 as lesion present.

Percentile-based 95% confidence intervals for all diagnostic performance and agreement estimates were obtained using a non-parametric cluster bootstrap with 10,000 replications at the horse level. Horses were resampled with replacement, with all observations from each selected horse retained together. Replications in which a statistic was not estimable were excluded from the percentile calculation for that statistic.

## 3. Results

Observer-specific CT and DT lesion-severity score distributions and majority-based binary CT classifications are presented in Table S2. Across all three observers, 71 of 384 CT assessments and 36 of 384 DT assessments were classified as lesion-positive (scores 1–3). Of the lesion-positive CT assessments, 37 were graded as mild, 30 as moderate, and 4 as severe. The corresponding numbers for DT were 24, 10, and 2, respectively.

In the primary analysis using the majority-based CT classification as the reference, DT sensitivity was 34.6%, 26.9%, and 23.1% for Observers 1, 2, and 3, respectively (Table 1). Specificity ranged from 94.1% to 96.1%, diagnostic accuracy from 80.5% to 83.6%, and Youden’s index from 19.2% to 30.7%. Cohen’s κ for agreement between each observer’s DT classification and the majority-based CT reference ranged from 0.249 to 0.377.

In the secondary analysis using each observer’s corresponding CT classification as the reference, DT sensitivity was 60.0%, 25.9%, and 20.8% for Observers 1, 2, and 3, respectively (Table 1). The corresponding specificities were 99.1%, 94.1%, and 95.2%; diagnostic accuracies were 93.0%, 79.7%, and 81.3%; and Youden’s indices were 59.1%, 20.0%, and 16.0%. The corresponding Cohen’s κ values for within-observer agreement between DT and CT classifications were 0.689, 0.247, and 0.207, respectively.

In the lesion-specific analysis using the majority-based CT classification as the reference, DT sensitivity for osteophytes was 58.3% (7/12), 41.7% (5/12), and 33.3% (4/12) for Observers 1, 2, and 3, respectively (Table 2). Sensitivity for SB changes was 14.3% (2/14) for each observer. All predefined SB assessment sites were included. No assessments of the proximal sesamoid bones or the dorsal border of the navicular bone were classified as positive by the majority-based CT reference. Therefore, these sites did not affect the sensitivity estimates. Table S3 reports TP, FN, FP and TN counts relative to the majority-based CT reference, by anatomical site and observer.

For osteophytes, Fleiss’ κ was 0.351 for DT and 0.400 for CT, with corresponding Gwet’s AC1 values of 0.593 and 0.507, respectively. For SB changes, Fleiss’ κ was 0.055 for DT and 0.454 for CT, with corresponding Gwet’s AC1 values of 0.925 and 0.838, respectively. Lesion-specific agreement estimates and their 95% confidence intervals are presented in Table 3.

## 4. Discussion

DT demonstrated high specificity, whereas sensitivity was limited and varied among the three observers for detecting osteophytes and SB changes in the equine DIP and MCP joints when CT classifications were used as the reference. The same pattern was observed in the primary majority-based analysis and in the secondary observer-specific analysis. Consequently, DT produced relatively few false-positive classifications compared with CT but missed a large proportion of CT-positive assessments. Because CT-negative assessments predominated under both reference approaches, diagnostic accuracy was strongly influenced by the large number of true-negative DT classifications and should therefore be interpreted alongside sensitivity and Youden’s index. The overall diagnostic performance estimates were derived from the predefined assessment set, comprising two joint-level osteophyte assessments and six site-level SB assessments per limb. Because these sites differed in anatomical location and in the frequency of CT-positive findings, the overall estimates depend on the composition of the assessment set and should not be interpreted as diagnostic performance for a specific joint or anatomical site. These results are consistent with those of the only previous equine cadaveric study investigating DT, in which DT was comparable to radiography, whereas volumetric imaging provided a more detailed evaluation of SB and periarticular margins [28]. That study also demonstrated that DT was capable of detecting a radiographically occult SB lesion [28]. The present findings do not support the use of DT as a replacement for CT for the comprehensive assessment of osseous joint pathology. Nevertheless, DT may have potential as a complementary imaging modality in selected clinical situations, but its clinical value was not assessed in the present study. Because conventional radiography was not included, the present study cannot determine whether DT provides additional diagnostic information beyond radiography.

Under the majority-based CT reference, sensitivity was numerically higher for osteophytes than for SB changes for all three observers. No formal statistical comparison between lesion categories was performed. The two lesion categories also comprised different anatomical assessments and differed in the frequency of CT-positive findings. The numerical differences between their sensitivity estimates should therefore be interpreted descriptively.

Although direct comparison is limited by differences in species and anatomical region, a human cadaveric hand study using CT as the reference also reported higher DT sensitivity for osteophytes than for subchondral sclerosis or subchondral cysts [37]. One possible explanation for the numerically higher sensitivity estimates for osteophytes is their location at the joint margins, where alteration of the normal bone contour facilitates recognition. By contrast, SB changes are located within the bone immediately beneath the articular surface and may appear only as subtle changes in radiographic opacity, internal bone structure, or articular surface contour. Their detection may therefore depend more strongly on lesion size and contrast. However, lesion size, location and morphology were not evaluated as potential determinants of detection, so this explanation could not be tested in the present study. DT reduces anatomical superimposition, but blurred out-of-plane structures can reduce the contrast of structures within the focal plane [26]. This is particularly relevant for the detection of small intraosseous lesions, whereas marginal osteophytes may remain more readily visible.

CT enables detailed assessment of osseous abnormalities because of its higher contrast resolution and absence of anatomical superimposition [17,21]. A recent study investigating equine distal tarsal OA used CT as the reference imaging modality [17]. Radiographic scores were significantly lower than CT scores for several OA features, particularly SB radiolucencies. Radiographic sensitivity was generally low to moderate for most assessed OA features. Similarly, in equine MCP joints, CT yielded higher scores than radiography for osteophytes and SB lysis [19]. These findings support the selection of CT as a clinically relevant imaging comparator for evaluating the diagnostic performance of DT in the present study. Gross pathological assessment has been used for comparison with imaging findings in studies evaluating articular cartilage defects [38,39]. However, gross pathological or histopathological validation of the osteophytes and SB changes evaluated in the present study was not performed because the limbs were subsequently used in a separate study. Diagnostic performance was therefore assessed relative to CT classifications rather than absolute lesion status, and CT should not be regarded as a gold standard.

The primary majority-based analysis used the same CT reference classification for all observers, allowing a more direct comparison among observers and reducing the influence of any single CT observer. However, because the reference was derived from the CT classifications of the same three observers, it was not independent of the observers whose DT classifications were evaluated. In the secondary observer-specific analysis, each observer’s DT classifications were compared with their own CT classifications. Consequently, variation in these estimates may reflect differences in the interpretation of both CT and DT.

Standing CT has been used clinically to image the equine distal limb regions in sedated horses using cone-beam and helical systems [22,23,40,41]. These studies reported that examinations of diagnostic quality could be obtained, although motion artefacts sometimes required repeated acquisitions [22,23]. CT examination of the equine distal limb therefore does not necessarily require general anaesthesia, which should be considered when assessing the potential clinical role of DT.

In human musculoskeletal imaging, DT has demonstrated greater sensitivity than conventional radiography for detecting rheumatoid erosions and osteophytes [30,31,33]. DT also performed comparably to CT for detecting scaphoid fractures in a small cadaveric study [42]. The sensitivity estimates from the secondary observer-specific analysis were lower than those reported in these human applications. However, differences in species, anatomical region, target abnormalities and study design limit direct comparison.

In the secondary observer-specific analysis, performance varied considerably, with sensitivity ranging from 20.8% to 60.0% and Cohen’s κ values for agreement between DT and CT classifications within individual observers ranging from 0.207 to 0.689. Because each observer’s DT classification was evaluated against their own CT classification, the observed variability may reflect differences in the interpretation of both modalities. In a human study, an experienced reader achieved higher sensitivity for detecting osteophytes on tomosynthesis images than an inexperienced reader [43]. In the present study, the observers differed in their previous imaging experience and had only a limited number of specimens available for familiarisation with DT. These factors may have been relevant to the observed variation, but their effects were not formally evaluated. The limited-angle tomographic acquisition and residual blur from out-of-plane structures reduce lesion conspicuity compared with fully volumetric CT datasets and may therefore have contributed to the variability in observer performance [26,27,44].

Cohen’s κ was used to assess agreement between each observer’s DT classifications and both the corresponding observer-specific and majority-based CT classifications. Interobserver agreement was assessed separately for CT and DT using Fleiss’ κ and Gwet’s AC1. For both osteophytes and SB changes, Fleiss’ κ was numerically higher for CT, while Gwet’s AC1 was numerically higher for DT. For SB changes on DT, Fleiss’ κ was 0.055 and Gwet’s AC1 was 0.925. For osteophytes, the horse-cluster bootstrap confidence intervals overlapped between CT and DT for both coefficients. For SB changes, the intervals overlapped for Gwet’s AC1 but not for Fleiss’ κ. No formal statistical comparison of interobserver agreement between the modalities was performed. Most ratings indicated that the assessed finding was absent, and the two coefficients calculate the agreement expected by chance differently. 

Several additional limitations should be considered when interpreting the present findings. Although 128 anatomical assessments were evaluated per observer and modality, these assessments originated from 16 limbs from only nine horses. To account for the dependence of limbs and anatomical assessments within horses, 95% confidence intervals were estimated using a horse-level cluster bootstrap in which all observations from each selected horse were retained together. However, only nine horse clusters were available, and there were few CT-positive assessments in several observer and lesion categories. This limited the precision of the estimates and resulted in wide confidence intervals. Severe scores were rare, and there were too few moderate and severe findings for a reliable severity-specific analysis. Because CT and DT datasets were assessed in a mixed randomised sequence without a predefined washout period, observers may have recalled findings from one modality when assessing the other. The study was performed using cadaver limbs. This allowed standardised image acquisition under controlled conditions and avoided motion artefacts. However, the simulated weight-bearing condition consisted of static loading of isolated limbs and did not reproduce physiological weight-bearing in a living horse. The absence of pathological validation, the dependence of both CT reference approaches on the participating observers, and the lack of direct comparison with conventional radiography further limit the interpretation of the findings. Finally, the present study focused on osteophytes and SB changes associated with OA. Whether DT provides similar diagnostic performance for other articular or periarticular abnormalities, such as enthesophytes, cartilage lesions or periarticular mineralisation, warrants further investigation.

## 5. Conclusions

The sensitivity of DT for osteophytes and SB changes relative to CT classifications was limited and varied among observers, and DT did not identify a substantial proportion of CT-positive assessments. The same pattern was observed in the primary majority-based analysis and the secondary observer-specific analysis. Under the majority-based CT reference, sensitivity was numerically lower for SB changes than for osteophytes for all three observers. The present results describe the diagnostic performance of DT in cadaver limbs under controlled, simulated weight-bearing conditions. Evaluation in live horses and direct comparison with conventional radiography are needed to determine its clinical performance, potential additional diagnostic value, and role in equine orthopaedic imaging.

## Figures and Tables

**Table 1:** Diagnostic performance of DT relative to majority-based (primary analysis) and observer-specific (secondary analysis) CT references. Each row includes 128 assessments. *n*, number of assessments; TP, true positive; TN, true negative; FP, false positive; FN, false negative; CI, confidence interval.

CT Reference	Observer	TP (*n*)	TN (*n*)	FP (*n)*	FN (*n*)	Sensitivity (%) (95% CI)	Specificity (%) (95% CI)	Accuracy (%) (95% CI)	Youden’s Index (%) (95% CI)	Cohen’s κ (95% CI)
Majority-based CT	Observer 1	9	98	4	17	34.6 (13.8–56.0)	96.1 (92.3–100.0)	83.6 (78.7–89.2)	30.7 (9.9–52.1)	0.377 (0.134–0.586)
	Observer 2	7	96	6	19	26.9 (15.4–41.4)	94.1 (89.8–97.8)	80.5 (74.1–86.8)	21.0 (9.4–35.3)	0.259 (0.118–0.409)
	Observer 3	6	98	4	20	23.1 (9.5–39.3)	96.1 (92.8–99.0)	81.3 (74.2–87.5)	19.2 (5.6–34.4)	0.249 (0.077–0.411)
Observer-specific CT	Observer 1	12	107	1	8	60.0 (33.3–90.0)	99.1 (97.2–100.0)	93.0 (88.2–97.7)	59.1 (32.5–88.8)	0.689 (0.435–0.894)
Observer-specific CT	Observer 2	7	95	6	20	25.9 (15.4–36.0)	94.1 (89.7–97.8)	79.7 (75.0–84.2)	20.0 (9.3–29.3)	0.247 (0.118–0.351)
Observer-specific CT	Observer 3	5	99	5	19	20.8 (6.3–32.1)	95.2 (91.2–99.0)	81.3 (77.3–84.6)	16.0 (1.3–26.7)	0.207 (0.018–0.328)

**Table 2:** Sensitivity of DT for osteophytes and SB changes relative to the majority-based CT reference. *n/N*, number of true-positive DT assessments/number of CT-positive assessments; CI, confidence interval.

Finding	Observer	*n/N*	Sensitivity (%)	95% CI
Osteophytes	1	7/12	58.3	28.6–100.0
	2	5/12	41.7	14.3–80.0
	3	4/12	33.3	7.1–66.7
SB changes	1	2/14	14.3	0.0–29.4
	2	2/14	14.3	0.0–38.5
	3	2/14	14.3	0.0–33.3

**Table 3:** Interobserver agreement among three observers for binary classifications of osteophytes and subchondral bone changes on CT and DT. CT, computed tomography; DT, digital tomosynthesis; SB, subchondral bone; *n*, number of predefined anatomical assessments rated by all three observers; CI, confidence interval.

Finding	Imaging Modality	Fleiss’ κ (95% CI)	Gwet’s AC1 (95% CI)
Osteophytes (*n* = 32)	CT	0.400 (0.155–0.647)	0.507 (0.235–0.800)
	DT	0.351 (−0.005–0.617)	0.593 (0.429–0.729)
SB changes (*n* = 96)	CT	0.454 (0.277–0.628)	0.838 (0.773–0.892)
	DT	0.055 (−0.059–0.269)	0.925 (0.865–0.969)

## Data Availability

The anonymised observer-level CT and DT scores underlying the analyses, including horse and limb identifiers and a description of the variables and score coding, are provided in Supplementary Dataset S1.

## Supplementary Materials

The following supporting information can be downloaded at https://www.mdpi.com/article/10.3390/ani16182971/s1. Table S1: Signalment, limb specimens, and recorded clinical history of the horses included in the study; Table S2: Distribution of observer-specific CT and DT lesion-severity scores and majority-based binary CT classifications by joint and anatomical target; Table S3: Majority-based CT reference and paired DT results by anatomical site and observer; Supplementary Dataset S1: Anonymised original CT and DT scores assigned by all three observers for each limb and anatomical assessment target, including horse and limb identifiers and a description of the variables and score coding.

## Abbreviations

The following abbreviations are used in this manuscript:

CT	Computed tomography
DIP	Distal interphalangeal
DT	Digital tomosynthesis
FN	False negative
FP	False positive
MC3	Third metacarpal bone
MCP	Metacarpophalangeal
OA	Osteoarthritis
P1	Proximal phalanx
P2	Middle phalanx
P3	Distal phalanx
SB	Subchondral bone
SWB	Simulated weight-bearing
TN	True negative
TP	True positive
