# Supplementary material for: Diagnostic Performance of Digital Tomosynthesis for Detecting Osteophytes and Subchondral Bone Changes in the Equine Distal Interphalangeal and Metacarpophalangeal Joints
Source: Animals (Basel). 2026 Sep 21;16(18):2971. doi: 10.3390/ani16182971 (PMC13603353; doi:10.3390/ani16182971)
Supplement: Supplementary file 1 [file animals-16-02971-s001.zip › Supplementary Material_Animals_Hahn.pdf]

Diagnostic Performance of Digital Tomosynthesis for Detecting Osteophytes and Subchondral Bone Changes in the Equine Distal Interphalangeal and Metacarpophalangeal Joints

Alisa Hahn [1,*], Miguel Valdés Vázquez [2], Gabriel Manso-Díaz [3], Dagmar Berner [4], Joana Maia Ramos [2], Marcus G. Doherr [5] and Christoph J. Lischer [1]

[1] Equine Clinic, Veterinary Hospital, School of Veterinary Medicine, Freie Universität Berlin, Oertzenweg 19b, 14163 Berlin, Germany; christoph.lischer@fu-berlin.de
[2] Hospital de Referencia La Equina, Apdo. 110, Camino de Martagina Km 1, 29692 Manilva, Málaga, Spain; miguel@laequina.com (M.V.V.); joana.126@hotmail.com (J.M.R.)
[3] Department of Animal Medicine and Surgery, Faculty of Veterinary Medicine, Complutense University of Madrid, Avenida Puerta de Hierro, s/n, 28040 Madrid, Spain; gmanso@ucm.es
[4] Equine Referral Hospital, Department of Clinical Science and Services, Royal Veterinary College, Hawkshead Lane, North Mymms, Hatfield, Hertfordshire AL9 7TA, UK; dberner@rvc.ac.uk
[5] Institute of Veterinary Epidemiology and Biostatistics, School of Veterinary Medicine, Freie Universität Berlin, Königsweg 67, 14163 Berlin, Germany; marcus.doherr@fu-berlin.de
* Correspondence: a.hahn@fu-berlin.de

Supplementary Material

Table S1. Signalment, limb specimens, and recorded clinical history of the nine horses included in the study. LF, left forelimb; RF, right forelimb.

Horse	Case number(s) and forelimb(s)	Age (years)	Sex	Breed	Body weight (kg)	Recorded clinical history
Horse 1	01 (RF)	20	Gelding	Holsteiner	500	Large-colon displacement and gaseous distension; suspected small-intestinal obstruction; euthanasia, surgery not authorised
Horse 2	02 (LF)<br>03 (RF)	15	Mare	Arabian	480	Caecal torsion; intraoperative euthanasia
Horse 3	04 (LF)	19	Gelding	Hanoverian	575	Severe enteritis; gastric rupture; euthanasia because of a poor prognosis
Horse 4	05 (RF)<br>06 (LF)	7	Stallion	German Sport Horse	570	Colon gaseous distension and gastric overload; suspected right-sided displacement; euthanasia, surgery not authorised
Horse 5	07 (RF)<br>08 (LF)	12	Gelding	Hanoverian	500	Small-intestinal obstruction; euthanasia, surgery not authorised
Horse 6	09 (RF)<br>10 (LF)	12	Mare	German Sport Horse	500	Colitis; suspected small-intestinal ileus; euthanasia because of a poor prognosis
Horse 7	11 (RF)<br>12 (LF)	3	Mare	Thoroughbred	480	Severe impaction of the ascending and descending colon; displacement of the ascending colon into the nephrosplenic space; postoperative colitis; euthanasia because of a poor prognosis
Horse 8	13 (RF)<br>14 (LF)	13	Gelding	Pura Raza Española	500	Suspected intoxication; haemolytic anaemia of unknown aetiology; euthanasia because of a poor prognosis
Horse 9	15 (RF)<br>16 (LF)	17	Mare	Thoroughbred	650	Large-colon gaseous distension, small-intestinal dilatation; horse was unable to rise due to severe neurological deficits; euthanasia because of a poor prognosis

Table S2. Distribution of CT and DT lesion-severity scores by anatomical target.

Panel A. Majority-based CT classifications and observer-specific DT lesion-severity scores.

Joint	Anatomical target	Imaging modality	Observer	Total assessments (n)	No lesion (n)	Mild (n)	Moderate (n)	Severe (n)	Lesion-positive assessments (n)
DIP joint	Osteophytes	CT	Majority of 3 observers	16	11	–	–	–	5
		DT	Observer 1	16	12	4	0	0	4
			Observer 2	16	8	7	1	0	8
			Observer 3	16	13	2	1	0	3
DIP joint	Subchondral bone changes, P2	CT	Majority of 3 observers	16	15	–	–	–	1
		DT	Observer 1	16	16	0	0	0	0
			Observer 2	16	16	0	0	0	0
			Observer 3	16	16	0	0	0	0
DIP joint	Subchondral bone changes, P3	CT	Majority of 3 observers	16	14	–	–	–	2
		DT	Observer 1	16	15	1	0	0	1
			Observer 2	16	16	0	0	0	0
			Observer 3	16	16	0	0	0	0
DIP joint	Subchondral bone changes, dorsal border of the navicular bone	CT	Majority of 3 observers	16	16	–	–	–	0
		DT	Observer 1	16	16	0	0	0	0
			Observer 2	16	16	0	0	0	0
			Observer 3	16	14	0	1	1	2

Panel A. Majority-based CT classifications and observer-specific DT lesion-severity scores (continued).

Joint	Anatomical target	Imaging modality	Observer	Total assessments (n)	No lesion (n)	Mild (n)	Moderate (n)	Severe (n)	Lesion-positive assessments (n)
MCP joint	Osteophytes	CT	Majority of 3 observers	16	9	—	—	—	7
		DT	Observer 1	16	11	4	1	0	5
			Observer 2	16	14	1	1	0	2
			Observer 3	16	13	1	2	0	3
MCP joint	Subchondral bone changes, P1	CT	Majority of 3 observers	16	12	—	—	—	4
		DT	Observer 1	16	16	0	0	0	0
			Observer 2	16	14	1	1	0	2
			Observer 3	16	14	1	0	1	2
MCP joint	Subchondral bone changes, MC3	CT	Majority of 3 observers	16	9	—	—	—	7
		DT	Observer 1	16	13	1	2	0	3
			Observer 2	16	15	1	0	0	1
			Observer 3	16	16	0	0	0	0
MCP joint	Subchondral bone changes, proximal sesamoid bones	CT	Majority of 3 observers	16	16	—	—	—	0
		DT	Observer 1	16	16	0	0	0	0
			Observer 2	16	16	0	0	0	0
			Observer 3	16	16	0	0	0	0

Panel A. Majority-based CT classifications and observer-specific DT lesion-severity scores (continued)

Joint	Anatomical target	Imaging modality	Observer	Total assessments (n)	No lesion (n)	Mild (n)	Moderate (n)	Severe (n)	Lesion-positive assessments (n)
**All joints**	**All anatomical targets**	**CT**	**Majority of 3 observers**	**128**	**102**	**—**	**—**	**—**	**26**
		**DT**	**Observer 1**	**128**	**115**	**10**	**3**	**0**	**13**
			**Observer 2**	**128**	**115**	**10**	**3**	**0**	**13**
			**Observer 3**	**128**	**118**	**4**	**4**	**2**	**10**

—, not applicable: no majority-based severity grade was assigned. Scores: 0, no lesion; 1, mild; 2, moderate; 3, severe; CT, computed tomography; DT, digital tomosynthesis; DIP, distal interphalangeal joint; MCP, metacarpophalangeal joint; P1, proximal phalanx; P2, middle phalanx; P3, distal phalanx; MC3, third metacarpal bone; n, number of assessments.

Panel B. Observer-specific CT and DT lesion-severity scores.

Joint	Anatomical target	Imaging modality	Observer	Total assessments (n)	No lesion (n)	Mild (n)	Moderate (n)	Severe (n)	Lesion-positive assessments (n)
DIP joint	Osteophytes	CT	Observer 1	16	9	4	3	0	7
			Observer 2	16	10	5	1	0	6
			Observer 3	16	14	1	0	1	2
		DT	Observer 1	16	12	4	0	0	4
			Observer 2	16	8	7	1	0	8
			Observer 3	16	13	2	1	0	3
DIP joint	Subchondral bone changes, P2	CT	Observer 1	16	16	0	0	0	0
			Observer 2	16	15	0	1	0	1
			Observer 3	16	14	1	1	0	2
		DT	Observer 1	16	16	0	0	0	0
			Observer 2	16	16	0	0	0	0
			Observer 3	16	16	0	0	0	0
DIP joint	Subchondral bone changes, P3	CT	Observer 1	16	16	0	0	0	0
			Observer 2	16	14	1	1	0	2
			Observer 3	16	12	1	3	0	4
		DT	Observer 1	16	15	1	0	0	1
			Observer 2	16	16	0	0	0	0
			Observer 3	16	16	0	0	0	0
DIP joint	Subchondral bone changes, dorsal border of the navicular bone	CT	Observer 1	16	16	0	0	0	0
			Observer 2	16	16	0	0	0	0
			Observer 3	16	16	0	0	0	0
		DT	Observer 1	16	16	0	0	0	0
			Observer 2	16	16	0	0	0	0
			Observer 3	16	14	0	1	1	2

Panel B. Observer-specific CT and DT lesion-severity scores (continued).

Joint	Anatomical target	Imaging modality	Observer	Total assessments (n)	No lesion (n)	Mild (n)	Moderate (n)	Severe (n)	Lesion-positive assessments (n)
MCP joint	Osteophytes	CT	Observer 1	16	8	6	2	0	8
			Observer 2	16	11	4	0	1	5
			Observer 3	16	11	4	0	1	5
		DT	Observer 1	16	11	4	1	0	5
			Observer 2	16	14	1	1	0	2
			Observer 3	16	13	1	2	0	3
MCP joint	Subchondral bone changes, P1	CT	Observer 1	16	15	0	1	0	1
			Observer 2	16	11	2	3	0	5
			Observer 3	16	11	3	2	0	5
		DT	Observer 1	16	16	0	0	0	0
			Observer 2	16	14	1	1	0	2
			Observer 3	16	14	1	0	1	2
MCP joint	Subchondral bone changes, MC3	CT	Observer 1	16	12	2	2	0	4
			Observer 2	16	8	2	6	0	8
			Observer 3	16	10	1	4	1	6
		DT	Observer 1	16	13	1	2	0	3
			Observer 2	16	15	1	0	0	1
			Observer 3	16	16	0	0	0	0
MCP joint	Subchondral bone changes, proximal sesamoid bones	CT	Observer 1	16	16	0	0	0	0
			Observer 2	16	16	0	0	0	0
			Observer 3	16	16	0	0	0	0
		DT	Observer 1	16	16	0	0	0	0
			Observer 2	16	16	0	0	0	0
			Observer 3	16	16	0	0	0	0

Panel B. Observer-specific CT and DT lesion-severity scores (continued)

Joint	Anatomical target	Imaging modality	Observer	Total assessments (n)	No lesion (n)	Mild (n)	Moderate (n)	Severe (n)	Lesion-positive assessments (n)
All joints	All anatomical targets	CT	Observer 1	128	108	12	8	0	20
			Observer 2	128	101	14	12	1	27
			Observer 3	128	104	11	10	3	24
		DT	Observer 1	128	115	10	3	0	13
			Observer 2	128	115	10	3	0	13
			Observer 3	128	118	4	4	2	10

Scores: 0, no lesion; 1, mild; 2, moderate; 3, severe; Lesion-positive assessments comprise scores 1-3. CT, computed tomography; DT, digital tomosynthesis; DIP, distal interphalangeal joint; MCP, metacarpophalangeal joint; P1, proximal phalanx; P2, middle phalanx; P3, distal phalanx; MC3, third metacarpal bone; *n*, number of assessments

Table S3. DT results relative to the majority-based CT reference by anatomical target and observer. The CT reference was positive when at least two of three observers assigned a score of 1–3 to the same limb and anatomical target. Each observer contributed 16 assessments per target and 128 overall. CT, computed tomography; DT, digital tomosynthesis; DIP, distal interphalangeal joint; MCP, metacarpophalangeal joint; MC3, third metacarpal bone; P1–P3, proximal, middle and distal phalanges, respectively; FN, false negative; FP, false positive; TN, true negative; TP, true positive; n, number of assessments.

Joint	Anatomical target	CT majority-positive (n)	CT majority-negative (n)	DT observer	TP (n)	FN (n)	FP (n)	TN (n)
**DIP joint**	Osteophytes	5	11	Observer 1	3	2	1	10
				Observer 2	3	2	5	6
				Observer 3	2	3	1	10
**DIP joint**	Subchondral bone changes, P2	1	15	Observer 1	0	1	0	15
				Observer 2	0	1	0	15
				Observer 3	0	1	0	15
**DIP joint**	Subchondral bone changes, P3	2	14	Observer 1	0	2	1	13
				Observer 2	0	2	0	14
				Observer 3	0	2	0	14
**DIP joint**	Subchondral bone changes, dorsal border of the navicular bone	0	16	Observer 1	0	0	0	16
				Observer 2	0	0	0	16
				Observer 3	0	0	2	14
**MCP joint**	Osteophytes	7	9	Observer 1	4	3	1	8
				Observer 2	2	5	0	9
				Observer 3	2	5	1	8
**MCP joint**	Subchondral bone changes, P1	4	12	Observer 1	0	4	0	12
				Observer 2	1	3	1	11
				Observer 3	2	2	0	12
**MCP joint**	Subchondral bone changes, MC3	7	9	Observer 1	2	5	1	8
				Observer 2	1	6	0	9
				Observer 3	0	7	0	9

Joint	Anatomical target	CT majority-positive (n)	CT majority-negative (n)	DT observer	TP (n)	FN (n)	FP (n)	TN (n)
**MCP joint**	Subchondral bone changes, proximal sesamoid bones	0	16	Observer 1	0	0	0	16
				Observer 2	0	0	0	16
				Observer 3	0	0	0	16
**All joints**	**All anatomical sites**	**26**	**102**	Observer 1	9	17	4	98
				Observer 2	7	19	6	96
				Observer 3	6	20	4	98
